# Rubella Virus: First Calcium-Requiring Viral Fusion Protein

**DOI:** 10.1371/journal.ppat.1004530

**Published:** 2014-12-04

**Authors:** Mathieu Dubé, Felix A. Rey, Margaret Kielian

**Affiliations:** 1 Department of Cell Biology, Albert Einstein College of Medicine, Bronx, New York, United States of America; 2 Unité de Virologie Structurale, Institut Pasteur and CNRS UMR 3569, Paris, France; North Carolina State University, United States of America

## Abstract

Rubella virus (RuV) infection of pregnant women can cause fetal death, miscarriage, or severe fetal malformations, and remains a significant health problem in much of the underdeveloped world. RuV is a small enveloped RNA virus that infects target cells by receptor-mediated endocytosis and low pH-dependent membrane fusion. The structure of the RuV E1 fusion protein was recently solved in its postfusion conformation. RuV E1 is a member of the class II fusion proteins and is structurally related to the alphavirus and flavivirus fusion proteins. Unlike the other known class II fusion proteins, however, RuV E1 contains two fusion loops, with a metal ion complexed between them by the polar residues N88 and D136. Here we demonstrated that RuV infection specifically requires Ca^2+^ during virus entry. Other tested cations did not substitute. Ca^2+^ was not required for virus binding to cell surface receptors, endocytic uptake, or formation of the low pH-dependent E1 homotrimer. However, Ca^2+^ was required for low pH-triggered E1 liposome insertion, virus fusion and infection. Alanine substitution of N88 or D136 was lethal. While the mutant viruses were efficiently assembled and endocytosed by host cells, E1-membrane insertion and fusion were specifically blocked. Together our data indicate that RuV E1 is the first example of a Ca^2+^-dependent viral fusion protein and has a unique membrane interaction mechanism.

## Author Summary

Rubella virus (RuV) is a small enveloped RNA virus causing mild disease in children. However, infection of pregnant women can produce fetal death or congenital rubella syndrome, a constellation of severe birth defects including cataracts, hearing loss, heart disease and developmental delays. While vaccination has greatly reduced disease in the developed world, rubella remains prevalent in developing countries and other undervaccinated populations. RuV infects cells by endocytic uptake and a low pH-triggered membrane fusion reaction mediated by the viral E1 protein. The postfusion structure of E1 revealed a metal ion complexed at the membrane-interacting tip of the protein. Here we demonstrated that RuV infection and fusion are completely dependent on calcium, which could not be replaced functionally by any other metal that was tested. In the absence of calcium, RuV entry and low pH-conformational changes were unchanged, but E1's interaction with the target membrane was specifically blocked. Mutations of the calcium-binding residues in E1 caused a similar inhibition of E1 membrane interaction, fusion and infection. Thus, RuV E1 is the first known example of a calcium-dependent virus fusion protein.

## Introduction

Rubella virus (RuV) is the causative agent of “German measles”, an airborne, relatively mild childhood disease [for review], [ see 1,2]. However, RuV infection of pregnant women can have very serious consequences due to transplacental infection of the fetus, causing first trimester miscarriages and severe fetal malformations known collectively as rubella congenital syndrome [2], [3]. Humans are the only known host for this virus. An efficient vaccine has all but eliminated the incidence of RuV disease in the Americas, although a reduction in vaccination recently led to resurgent cases [4]. RuV remains endemic worldwide and is a serious public health problem in countries without an effective vaccination program, which included 59% of the world birth cohort in 2012 [5].

RuV is the sole member of the *Rubivirus* genus, which together with the *Alphavirus* genus makes up the *Togaviridae* family. As for all togaviruses, RuV is an enveloped virus with a positive strand RNA genome [for overview, see 2]. RuV is roughly spherical but relatively pleomorphic, with diameters ranging from ∼60–80 nm [6]–[8]. The RuV genome is organized similarly to that of alphaviruses, and encodes the non-structural proteins p150 and p90, which mediate viral RNA replication and transcription, and the structural proteins capsid, E2, and E1. The genome associates with capsid proteins to form the internal nucleocapsid core, which is enveloped by a lipid bilayer containing heterodimers of the E2 and E1 transmembrane glycoproteins. While this general organization is similar to that of the alphaviruses, cryo-electron tomography studies show that the RuV surface is organized with parallel rows of E2-E1 heterodimers rather than the T = 4 icosahedral symmetry of the alphaviruses [7], [8].

During RuV biogenesis the structural polyprotein p110 is translated in association with the endoplasmic reticulum (ER), and signal peptidase cleavage produces the capsid, E2 and E1 proteins. E2 and E1 form stable heterodimers within the ER, with E2 acting as a chaperone to promote the folding and sorting of E1 [9]–[12]. Unlike alphavirus E2, which is processed by cellular furin to prime virus infectivity [13], RuV E2 and E1 do not undergo a proteolytic maturation step [2]. RuV particles bud into the Golgi apparatus, traffic through the secretory pathway, and are released into the extracellular milieu [14]–[16].

Most of the RuV antigenic determinants and all of its neutralizing epitopes are located on the E1 protein [17], [18], which is therefore postulated to mask the E2 protein in the virus particle. During RuV entry E1 binds to cell surface receptors [19] that mediate virus uptake by clathrin-dependent [20] and/or macropinocytic [21] pathways. Similar to the alphaviruses [22], RuV fuses in endosomes via low pH-triggered conformational changes in the E1 fusion protein [23]–[26]. However, in spite of this general outline of RuV entry, mechanistic information is relatively limited and is heavily based on analogy with the alphavirus entry and fusion pathways.

The alphavirus and flavivirus membrane fusion proteins have similar structures and have been grouped together as “class II” fusion proteins [reviewed in 27]. The postfusion structure of the RuV E1 ectodomain was recently determined [28], revealing it to be a class II fusion protein despite the lack of amino acid sequence conservation with the alpha- or flavivirus fusion proteins. Similar to other class II proteins, RuV E1 is composed of three β-sheet-rich domains: a central domain I (DI) connecting on one side to the elongated DII, and on the other to the Ig-like DIII followed by the stem and transmembrane (TM) regions [28]. As for the postfusion forms of other class II proteins, postfusion RuV E1 is a stable homotrimer with a central trimer core composed of DI and DII, against which the DIII and stem regions fold back to form an overall hairpin-like configuration. Extensive studies of class II proteins demonstrate that fusion is driven by the initial insertion of the fusion loop (FL) at the tip of DII into the target membrane, followed by refolding to the hairpin thus bringing the viral and target membranes together [reviewed in 27], [29].

In addition to these similarities, the structure of RuV E1 revealed a puzzling difference from other class II fusion proteins, and indeed from all other known viral fusion protein structures [28]. Rather than the single FL of other class II fusion proteins, RuV E1 contains two FLs at the tip of DII [30] (FL1, residues 88–93; FL2, residues 131–137), making a more extensive surface for target membrane interaction. Other fusion proteins such as those of the baculoviruses, herpesviruses, and rhabdoviruses have also been shown to contain two FLs [30]–[32]. However, in the case of RuV, a metal binding site is located between the two FLs. The E1 structure shows that this site coordinates either Na^+^ or Ca^2+^ ions via interactions with residues N88 in FL1 and D136 in FL2 [28]. Ca^2+^ binds with apparent higher affinity and displaces Na^+^. Ca^2+^ binding affects the conformation of FL2 around D136 with respect to the Na^+^ bound form, but does not cause other changes in E1. No viral fusion protein has ever been observed to require a metal ion for its function.

Here we addressed the role of Ca^2+^ in RuV infection. We found that Ca^2+^ was critical for productive viral entry. The absence of Ca^2+^ caused a block in E1-target membrane insertion and thus inhibited virus-membrane fusion. Similarly, alanine substitution of the Ca^2+^-coordinating residues N88 and D136 blocked virus membrane interaction, fusion and infection. Our observations thus document the first described example of a Ca^2+^-dependent viral fusion protein.

## Results

### Calcium is required during RuV infection

**Figure 1:**
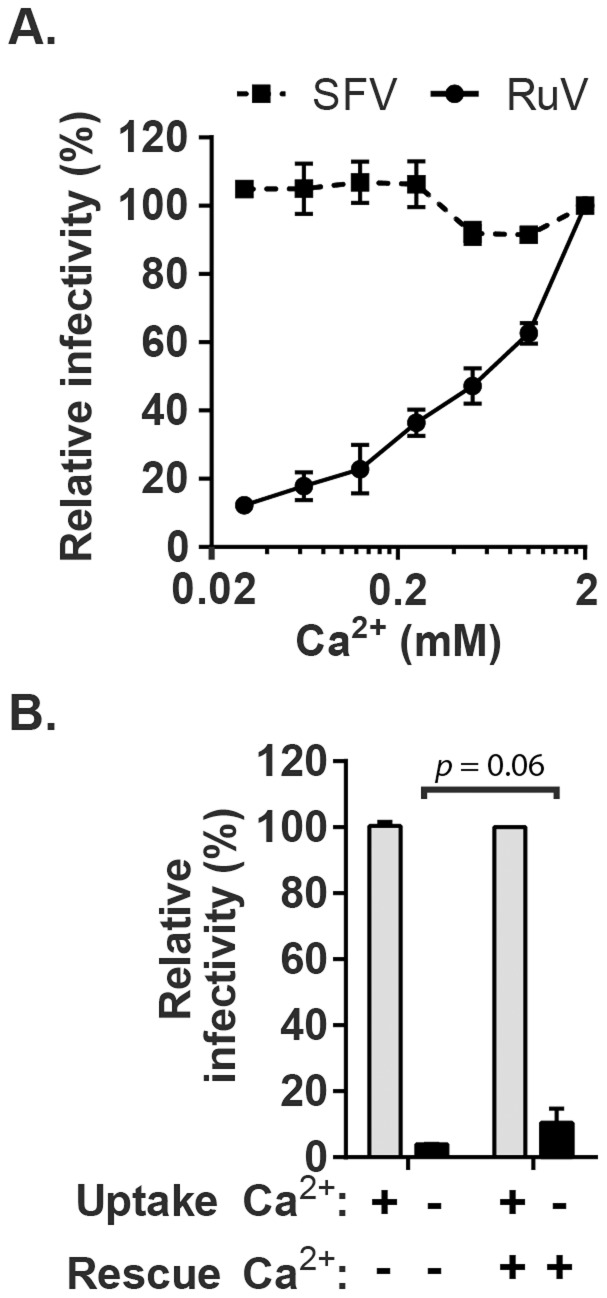
Effect of Ca^2+^ on RuV infection. (A) RuV or SFV was pre-bound to Vero cells on ice for 90 min in binding medium. Cells were shifted to 37°C for 20 min in medium containing the indicated concentrations of CaCl_2_, and then cultured for 48 h at 37°C in growth medium plus 20 mM NH_4_Cl to prevent secondary infection. Infected cells were scored by immunofluorescence. Infectivity was normalized to that observed at 2 mM CaCl_2_, which was ∼15% infected cells. (B) RuV was prebound to Vero cells as in panel A and incubated at 37°C for 20 min in medium with or without 2 mM CaCl_2_ (Uptake). The cells were then incubated for 1 h at 37°C in medium with or without 2 mM CaCl_2_, to test if infection could be rescued by the addition of Ca^2+^ (Rescue). The cells were then cultured for 48 h at 37°C in growth medium plus 20 mM NH_4_Cl, scored by immunofluorescence, and infectivity normalized to that observed when 2 mM CaCl_2_ was present throughout the experiment. Data in A are the mean and range of 2 independent experiments. Data in B are the mean and standard deviation from 3 independent experiments. Statistical analysis was performed in (B) using a paired Student's t test.

The importance of calcium during RuV entry was tested by pre-binding virus to target Vero cells on ice and then incubating at 37°C in medium containing various concentrations of CaCl_2_. To avoid possible effects of calcium deprivation on endosomal acidification [33], the internalization period was limited to 20 min. Cells were then incubated in growth medium containing NH_4_Cl to neutralize endosomal pH, and infected cells scored at 48 h post-infection by immunofluorescence (Fig. 1A). RuV infection was strongly dependent on the concentration of Ca^2+^, with infection efficiency decreased by more than 90% in the presence of 30 µM CaCl_2_ vs. 2 mM CaCl_2_. Infection by the low pH-dependent alphavirus Semliki Forest Virus (SFV) was independent of Ca^2+^ under these conditions, excluding aberrant endosomal acidification. Addition of CaCl_2_ after the 20 min internalization period but prior to endosomal neutralization by NH_4_Cl did not cause significant rescue of RuV infection (p>0.05) (Fig. 1B). This result suggests that the internalized RuV particles were inactivated by endosomal acidity during the 20 min uptake period. While the lack of rescue could be due to several factors such as rapid acid-inactivation and/or an inability of added calcium to access the virus-containing endosomes, it demonstrates that Ca^2+^ deprivation did not simply delay RuV endocytosis. Together our data support a critical and specific role of Ca^2+^ during productive RuV entry.

### Calcium is required for RuV fusion

**Figure 2:**
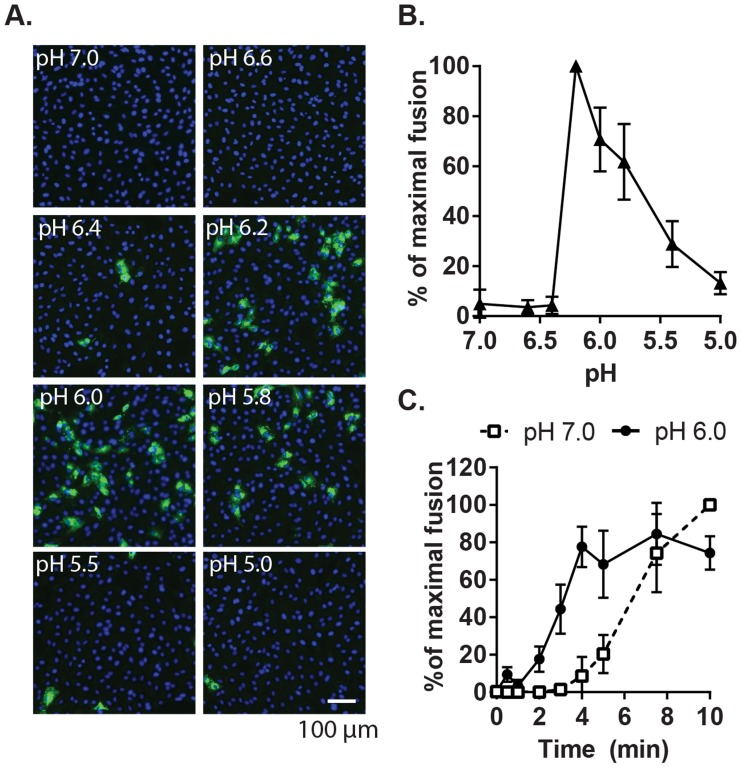
Characterization of the pH threshold for RuV fusion. (A) Fusion-infection assay. Virus was pre-bound to Vero cells as in Fig. 1A. Cells were washed and incubated for 3 min at 37°C in calcium-containing medium at the indicated pH, and then cultured 48 h at 37°C in growth medium plus 20 mM NH_4_Cl to prevent secondary infection. Infected cells were detected by immunofluorescence (green), and nuclei were counterstained with Hoescht (blue). (B) Quantitation of (A). Data were normalized to maximal fusion, which was observed at pH 6.2 in each experiment. Graph shows the mean and standard deviation of 4 independent experiments. (C) Kinetics of RuV fusion. A fusion infection assay was performed as in (A), using calcium-containing buffers at either pH 7.0 or pH 6.0 and treating for the indicated time at 37°C, followed by addition of growth medium containing 20 mM NH_4_Cl and culture for 48 h. Graph shows the mean and standard deviation of 3 independent experiments.

Given the location of bound Ca^2+^ between the two E1 FLs [28], we hypothesized that it plays a role in RuV fusion with target membranes, a complex process not yet fully understood. Although RuV is known to require acidification to initiate fusion [23], [24], its pH threshold has not been determined. A fusion-infection assay previously developed for alphaviruses [34] was used to measure this precisely. RuV was prebound to Vero cells on ice, pulsed for 3 min at 37°C with medium of defined pH, and the resultant infected cells scored by immunofluorescence (Fig. 2A and B). No infection was detected upon treatment at pH 7.0, indicating that virus fusion in endosomes does not occur within this time frame. Infection was observed after treatment at pH 6.2, and gradually decreased at lower pH values, presumably reflecting virus inactivation by acidic pH. The kinetics of RuV fusion were then determined by pulsing bound RuV at pH 7.0 or 6.0 for various times (Fig. 2C). Infection increased with time of treatment at pH 6.0, reaching a maximum at 4 min. Infection from pH 7.0 incubation was first detected after 5 min and increased thereafter, reflecting the time required for virus endocytic uptake and endosomal fusion. Together our data indicate that the RuV fusion reaction is optimal at 4 min of treatment at ∼pH 6.0–6.2 at 37°C.

**Figure 3:**
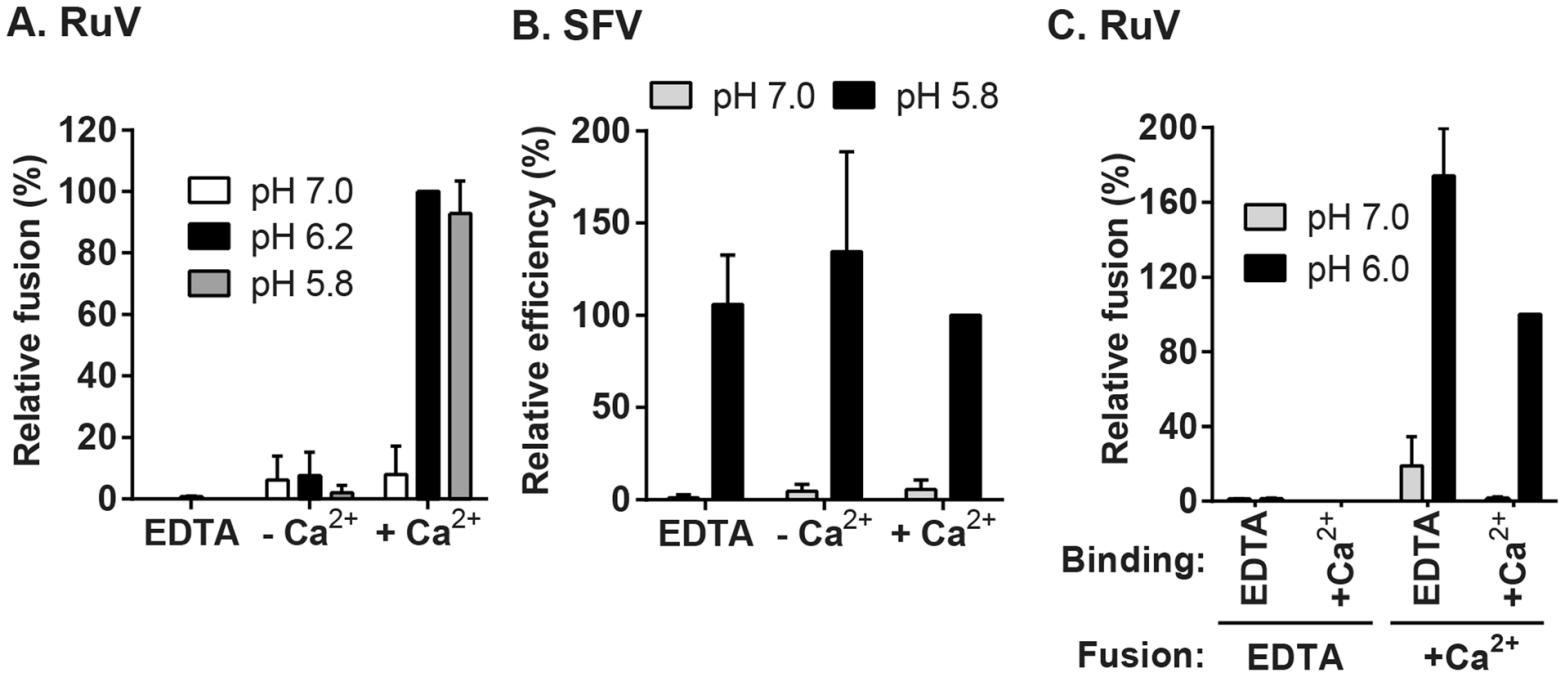
Effect of Ca^2+^ on RuV fusion. (A and B) Fusion-infection assays were performed with (A) RuV or (B) SFV as in Fig. 2A, but treating at the indicated pH for 4 min at 37°C in calcium-free fusion medium supplemented where indicated with 1.5 mM EDTA or 2 mM CaCl_2_. Data were normalized to the pH 6.2-treated samples in medium plus CaCl_2_, and are the mean and standard deviation of 3 independent experiments. (C) Modified fusion-infection assay in which RuV was pre-bound to Vero cells in binding buffer containing 1.5 mM EDTA or 2 mM CaCl_2_. Cells were then washed to remove unbound virus and treated for 4 min at 37°C with calcium-free fusion medium at the indicated pH, supplemented with either 1.5 mM EDTA or 2 mM CaCl_2_. Data were normalized to the pH 6.0 samples in which both the binding and fusion media contained calcium, and are the mean and range of 2 independent experiments.

We then used this assay to determine the role of Ca^2+^ in RuV fusion (Fig. 3A). Treatment for 4 min at neutral pH did not allow RuV fusion whether Ca^2+^ was present or not. In contrast, fusion at pH 6.0 or 5.8 was strongly dependent on Ca^2+^, with increases of up to 46-fold with 2 mM CaCl_2_ compared to calcium-free treatment. Addition of the calcium chelator EDTA completely abrogated fusion at both pH 5.8 and 6.0. Treatment at very low pH (5.0) did not rescue fusion in the absence of Ca^2+^ (Fig. S1), indicating that the fusion defect is not due to an acid-shift in the RuV pH threshold. A similar calcium dependence for RuV fusion was observed using HeLa cells or primary human umbilical vein endothelial cells as targets (Fig. S2). As predicted [34], [35], SFV fusion was strongly pH-dependent but calcium-independent (Fig. 3B).

To control for a possible effect of calcium on RuV binding to target cells, we performed fusion-infection assays in which the binding and/or fusion steps were carried out in the presence of EDTA or CaCl_2_ (Fig. 3C). As observed also in Fig. 3A, fusion was efficient when Ca^2+^ was present during both steps, and totally impaired when EDTA was included in the fusion media. However, even when RuV was prebound in presence of EDTA, it fused efficiently when subsequently treated at low pH in the presence of Ca^2+^. Together our data support a specific role for Ca^2+^ during virus fusion, and the lack of a Ca^2+^ requirement for virus-cell binding. Any effects of Ca^2+^ chelation on the virus were reversible by adding back Ca^2+^ during the fusion step.

### Neither Mg^2+^ nor acetate alters the RuV Ca^2+^ requirement

**Figure 4:**
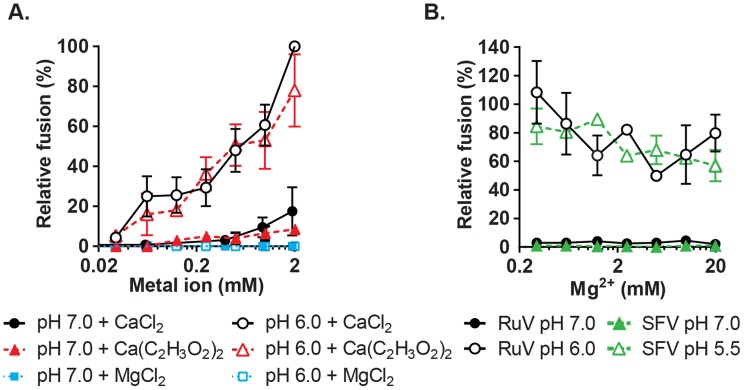
Concentration-dependence and specificity of RuV Ca^2+^ requirement. (A) RuV fusion infection assay was performed as in Fig. 3A, in the presence of CaCl_2_, MgCl_2_ or Ca(C_2_H_3_O_2_)_2_ buffered with 1 mM EGTA to produce the indicated concentrations of free Ca^2+^ or Mg^2+^. Data were normalized to the pH 6.0, 2 mM CaCl_2_ sample, and are the mean and standard deviation of 3 independent experiments. (B) RuV and SFV fusion infection assays were performed as in Fig. 3A, with the fusion media supplemented with 0.5 mM CaCl_2_ and 0.2–20 mM MgCl_2_. Data were normalized to pH 6.0 with 0.5 mM CaCl_2_ and no added MgCl_2_, and represent the mean and range of 2 independent experiments.

To more rigorously determine the Ca^2+^ concentration requirement for RuV fusion, we performed fusion-infection assays in which Ca^2+^ was buffered with EGTA, a more selective Ca^2+^ chelator. RuV fusion in this assay was maximal at 2 mM CaCl_2_ and showed a gradual reduction at decreasing Ca^2+^ concentrations (Fig. 4A). Mg^2+^, Mn^2+^ and Zn^2+^ did not substitute for Ca^2+^ even at concentrations of 2 mM (Fig. 4A, Fig. S3). When fusion was triggered using a sub-optimal concentration of Ca^2+^ (0.5 mM), the addition of Mg^2+^ at concentrations up to 20 mM neither substituted nor competed with Ca^2+^ (Fig. 4B). The structure of the RuV E1 homotrimer shows that the bound Ca^2+^ is coordinated in part by an acetate ion [28]. However, when tested in the fusion-infection assay CaCl_2_ and Ca(C_2_H_3_O_2_)_2_ promoted fusion with a similar concentration dependence (Fig. 4A), implying that acetate is not critical for the role of Ca^2+^ during fusion.

### Low pH-triggered E1 conformational changes are Ca^2+^ independent

**Figure 5:**
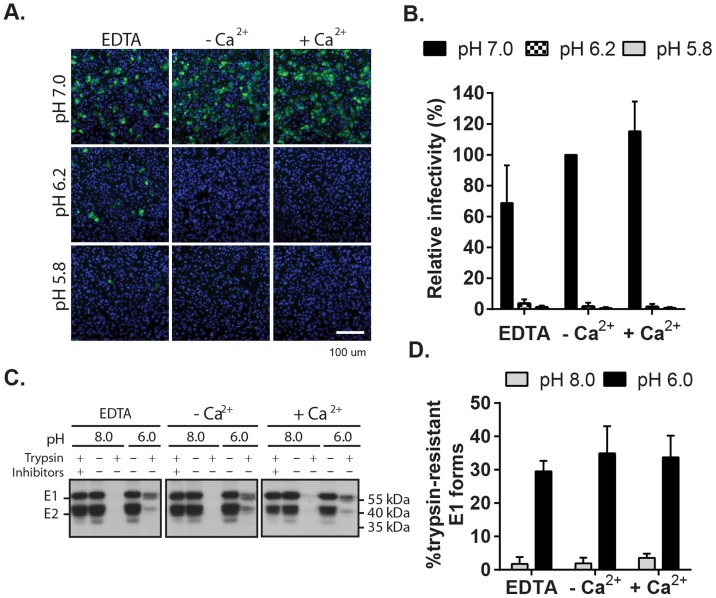
Impact of Ca^2+^ on RuV E1 low-pH triggered conformational change. (A) Low pH inactivation of RuV infectivity. RuV was adsorbed to poly-D-lysine coated wells and incubated for 15 min at 37°C in fusion media of the indicated pH and containing 2 mM CaCl_2_ or 1.5 mM EDTA where indicated. The wells were then washed, overlaid with Vero cells, incubated for 1 h at 37°C in complete medium with calcium to permit virus entry, and cultured for 48 h in presence of NH_4_Cl. Nuclei were stained with Hoescht and infection was quantitated by immunofluorescence. (B) Quantitation of data from (A). Infectivity was calculated relative to the control treated with buffer at pH 7.0. Error bars represent the standard deviation from the mean of 3 independent experiments. (C–D) E1 trypsin-resistance assay. (C) Purified radiolabeled RuV was treated at the indicated pH for 5 min at 37°C in the presence of 2 mM CaCl_2_ or 1.5 mM EDTA as indicated. Samples were digested with trypsin with or without inhibitors, and the E1 protein analyzed by immunoprecipitation and SDS-PAGE. (D) Quantitation of E1 trypsin resistance assays performed as in (C). Graph shows the mean and standard deviation of 3 independent experiments.

Low pH triggers the irreversible rearrangement of the prefusion RuV E2-E1 heterodimer to the E1 hairpin-like homotrimer, driving fusion of the virus with a target membrane [24], [28] or virus inactivation in the absence of target membranes [24]. To determine if the role of Ca^2+^ in fusion is via effects on this conformational change, we first examined the effect of Ca^2+^ on low pH inactivation [36]. RuV particles were immobilized on poly-D-lysine-coated wells, treated with neutral or low pH buffers in the presence or absence of CaCl_2_ or EDTA, overlaid with Vero cells and incubated for 48 h in growth media to quantitate virus infectivity (Fig. 5A and B). Fluorescence microscopy confirmed that buffer treatments did not cause elution of the adsorbed virus from the wells (Fig. 5A, Fig. S4). Virus infectivity was maintained after treatment at neutral pH whether or not Ca^2+^ or EDTA was present, but low pH caused virus inactivation under either condition.

To directly test the role of Ca^2+^ in the RuV E1 conformational change, we used a previously described assay [24] to compare the trypsin-resistance of prefusion vs. postfusion E1. Radiolabeled RuV was treated at neutral vs. low pH, solubilized in non-ionic detergent, digested with trypsin, and precipitated with a monoclonal antibody (mAb) to E1 (Fig. 5C and D). Non-digested samples treated at either pH showed efficient co-precipitation of the E2-E1 dimer. While trypsin completely digested the samples treated at pH 8.0, three distinct species were immunoprecipitated from the trypsinized low pH-treated samples: a tight doublet migrating slightly faster than uncleaved E1 (∼55 kDa) and a band of lower molecular weight (∼41 kDa). These species were immunoprecipitated by mAb to the E1 protein but not the E2 protein (Fig. S5), confirming that they were derived from digestion of the E1 protein. Low pH-triggered production of trypsin-resistant E1 was unaffected by the presence of either CaCl_2_ or EDTA. Thus, Ca^2+^ is not required for the low pH-triggered conformational change that leads to RuV inactivation and E1 trypsin-resistance.

### Ca^2+^ is required for RuV E1-membrane interaction

**Figure 6:**
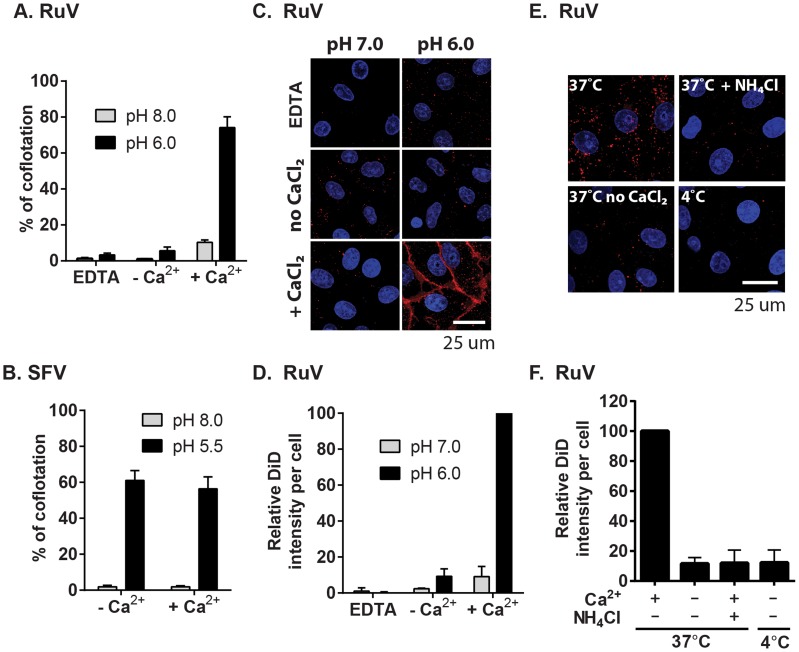
Calcium dependence of RuV-membrane interactions. (A–B) Virus-liposome binding assays. Radiolabeled RuV (A) or SFV (B) was incubated with liposomes for 1 min at 37°C at the indicated pH and buffer conditions. The percentage of total virus radioactivity associated with the liposomes was determined by sucrose gradient floatation. (C–D) Calcium requirement for virus fusion at the plasma membrane. (C) Purified RuV particles were labeled with DiD (red), prebound to Vero cells on ice, and the cells were pulsed at 37°C for 2 min in fusion medium containing 2 mM CaCl_2_ or 1.5 mM EDTA as indicated. Cells were then fixed and nuclei were counterstained with Hoescht (blue). Images represent a maximal projection of Z-stacks, with the scale bar indicating 25 µm. (D) Quantitation of C. DiD signal and cell numbers were quantitated and normalized to control, calcium-containing conditions. (E–F) Calcium requirement for virus fusion in endosomes. DiD-labeled virus was prebound to Vero cells on ice, and the cells with bound virus were incubated at 37°C for 20 min in binding medium containing 2 mM CaCl_2_ or 20 mM NH_4_Cl as indicated. Cells were then fixed, imaged and quantitated as in (C–D). Graphs show the mean and standard deviation of 3 (A, F) or 4 (D) independent experiments. For (B), the mean and range were calculated from 2 independent experiments.

An early step in viral membrane fusion pathways is the insertion of the fusion peptide or fusion loops into the target membrane. This step confers virus-target membrane association and can be monitored by testing the cofloatation of virus with target liposomes [37]. To test the role of Ca^2+^ in E1-membrane insertion, radiolabeled RuV was mixed with liposomes and treated at neutral or low pH in the presence of EDTA or CaCl_2_. Liposomes plus associated virus were separated on sucrose floatation gradients (Fig. 6A). Little virus was associated with the floated liposomes when treated at either pH in the absence of Ca^2+^. However, efficient virus-liposome interaction (∼74% of input virus) was observed when low pH treatment was carried out in the presence of 2 mM CaCl_2_. Even at pH 8.0, 2 mM CaCl_2_ caused a small increase (∼10%) in virus-liposome association. Once the virus was associated with liposomes, stable interaction was maintained even though the gradients did not contain Ca^2+^. Ca^2+^ did not promote non-specific electrostatic interactions of virus with membranes, since SFV-liposome floatation was strictly low pH-dependent and unaltered by the presence or absence of CaCl_2_ (Fig. 6B).

Our data indicate that complete RuV fusion is Ca^2+^-dependent. To test whether initial lipid mixing between RuV and cell membranes can occur in the absence of calcium, we labeled RuV with a self-quenching concentration of the lipophillic dye DiD. Virus was prebound to Vero cells at 4°C, and then samples were pulsed for 2 min at 37°C with medium at pH 6.0 or 7.0 and containing 2 mM CaCl_2_ or EDTA as indicated. Lipid mixing was assessed by measuring the increase in fluorescence that resulted from the dequenching of the DiD probe (Fig. 6C and 6D). Upon pulsing at pH 7.0, only background fluorescence was observed. In contrast, a strong homogeneous signal was detected at the surface of the cells after treatment at pH 6.0 in presence of CaCl_2_. This low pH-triggered fusion was blocked by omission of CaCl_2_ or the addition of EDTA. We then tested whether calcium was also required for fusion and lipid mixing in endosomes. DiD-labeled RuV was prebound to Vero cells and virus uptake allowed to occur for 20 min at 37°C in medium containing 2 mM CaCl_2_ or 20 mM NH_4_Cl. Cells were immediately fixed after the internalization period and analyzed by confocal microscopy. Virus fusion in endosomes resulted in numerous bright fluorescent foci when uptake occurred in presence of CaCl_2_ (Fig. 6E–D). DiD dequenching was severely impaired by preventing endosomal acidification with NH_4_Cl, by preventing endocytic uptake by incubation at 4°C, or by removing calcium from the media during virus internalization. Together, our data support a critical role of calcium for RuV E1-membrane interaction and for the initial lipid-mixing stage of fusion.

### Mutation of the Ca^2+^-coordinating residues on E1 specifically abrogates RuV infectivity

**Figure 7:**
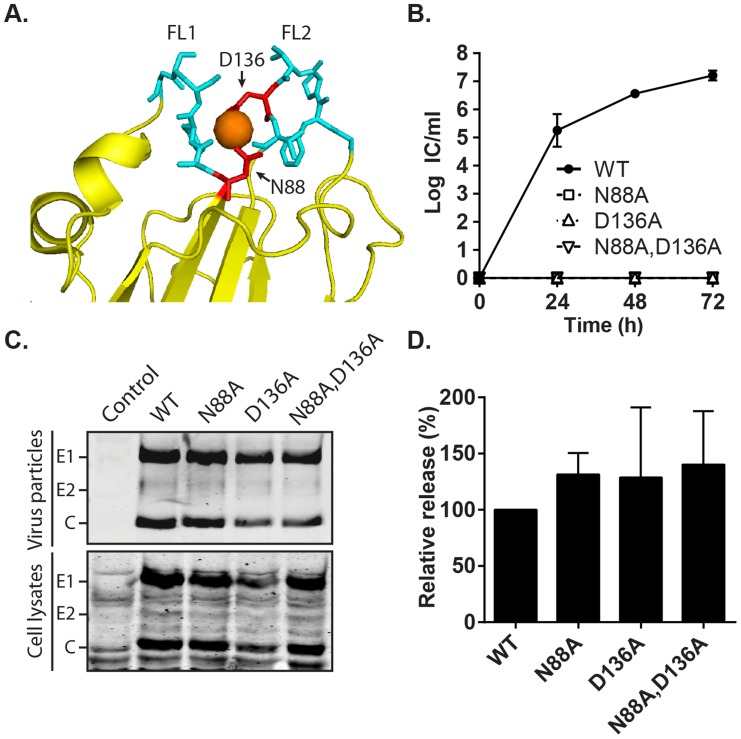
Effect of mutation of the RuV E1 Ca^2+^-coordinating residues on virus replication. (A) Close-up view of the tip of RuV E1 domain II [28] (PDB 4B3V). The two fusion loops are shown in cyan, the bound Ca^2+^ cation as an orange sphere, and the Ca^2+^-coordinating residues D136 and N88 in red. (B) Growth kinetics. BHK-21 cells were electroporated with the indicated viral RNAs, the growth medium collected at the indicated time points, and the progeny virus quantitated by infectious center (IC) assays. Data shown are the mean and range of 2 independent experiments. (C–D) Assembly assays. BHK-21 cells were electroporated with the indicated viral RNAs, and the growth medium and cell lysates harvested at 48 h. Virus particles in the medium and viral proteins in the lysates were visualized by SDS-PAGE and Western blot. (D) Quantitation of assembly assays performed as in (C). Graphs show the mean and standard deviation of 3 independent experiments, with release normalized to that of WT RuV.

The RuV E1 trimer structure shows that Ca^2+^ is coordinated by residues N88 and D136 in FL 1 and 2, respectively [28] (Fig. 7A). Such polar/charged residues are commonly involved in coordination of Ca^2+^, but are usually absent from fusion peptides [38], having the potential to hinder their insertion into the target membrane. We tested whether we could overcome the RuV calcium requirement by substituting these residues with alanine, an uncharged residue that is common in fusion loops [38]. Mutant viral RNAs were transcribed from the RuV infectious clone and electroporated into BHK-21 cells. Cells electroporated with WT RuV RNA produced infectious particles as soon as 24 h, and reached a maximum titer of 1×10^7^ IC/ml at 72 h post-electroporation (Fig. 7B). In contrast, neither the single N88A or D136A mutants nor the double mutant N88A,D136A produced detectable infectious virus under the same conditions. Equivalent expression of E1, E2 and capsid was detected in WT vs. mutant-infected cells, ruling out a problem in mutant viral protein expression (Fig. 7C). The amount of virus particles pelleted from the medium at 48 h post-electroporation was comparable between WT and mutants (Fig. 7C). The efficiency of particle release (normalized to expression levels in the cell lysates) was equivalent between WT and mutants (Fig. 7D). Thus, mutation of the Ca^2+^-coordinating residues in RuV E1 does not overcome the requirement for calcium. Instead, these mutations are lethal to RuV infection without affecting virus synthesis and assembly.

### Phenotype of the E1 N88A,D136A mutant recapitulates that observed in absence of Ca^2+^

**Figure 8:**
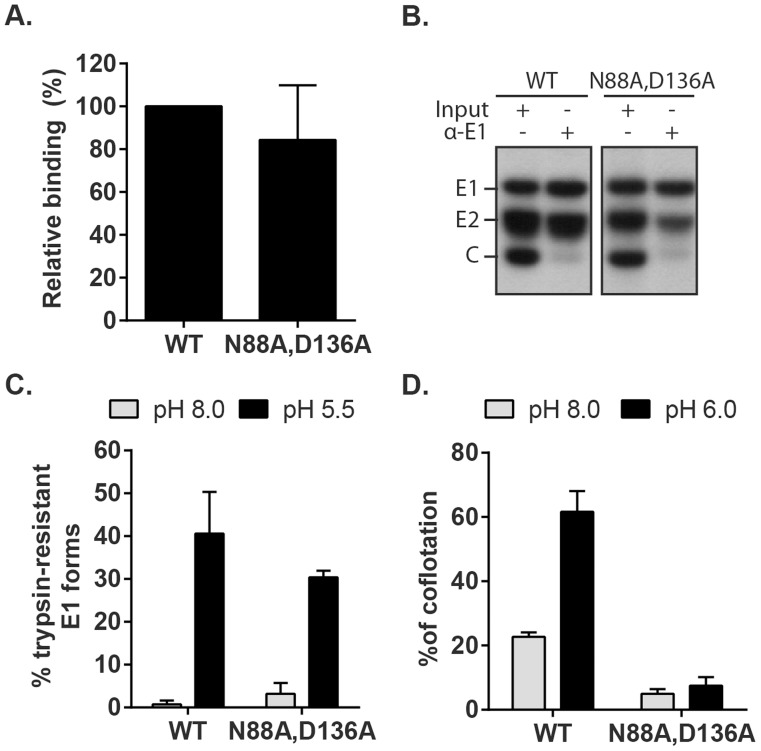
Characterization of RuV E1 N88A,D136A virus. (A) Cell binding. Radiolabeled WT RuV or N88A,D136A mutant was bound to Vero cells on ice. Binding efficiency was calculated relative to WT RuV. (B) Stability of the RuV E2-E1 heterodimer. Radiolabeled WT RuV or N88A,D136A mutant viruses were lysed in detergent, E1 was immunoprecipitated, and co-retrieval of E2 was determined by SDS-PAGE and autoradiography. (C) E1 trypsin resistance. Radiolabeled WT RuV or N88A,D136A mutant was treated for 1 min at 37°C and the indicated pH, and E1 trypsin-resistance quantitated as in Fig. 5C. (D) Virus-liposome association. Liposome association of radiolabeled WT RuV or N88A,D136A mutant was determined as in Fig. 6A in presence of 2 mM CaCl_2_. Graphs show the mean and standard deviation of 5 (A) or 3 (C and D) independent experiments.

Fusion-infection assays of the mutants revealed that their infectivity was not rescued even by low pH-treatment in the presence of 20 mM CaCl_2_ (Fig. S6). We therefore tested specific steps of entry and fusion for the N88A,D136A double mutant. Binding studies showed that the efficiency of radiolabeled WT and mutant virus binding to Vero cells on ice was comparable (Fig. 8A). Co-immunoprecipitation analysis of solubilised virus showed that both the WT and mutant E1 proteins were efficiently recognized by a commercial mAb to RuV E1 and that similar levels of E2 were retrieved, indicating comparable stability of the E2-E1 dimer interaction (Fig. 8B). Together with the efficient assembly of the mutant (Fig. 7D), these results thus suggest that the folding of N88A,D136A E1 is not aberrant.

To test the possibility that these mutations compromise the low pH-triggered structural reorganization of the E1 protein, we evaluated the conversion of E1 N88A,D136A to trypsin-resistance (Fig. 8C). Both WT and mutant E1 were totally digested by trypsin after treatment at neutral pH, consistent with a similar heterodimeric structure. After low pH treatment similar proportions of the mutant and E1 WT were trypsin-resistant (Fig. 8C). We then compared the membrane interaction of WT virus and the N88A,D136A mutant (Fig. 8D). While low pH treatment in the presence of Ca^2+^ produced efficient liposome association of the WT virus, the mutant virus showed only background levels of liposome floatation at either pH. Thus, our data indicate that the N88A,D136A mutant virus was comparable to the WT in all properties save for the ability of mutant E1 to interact with the target bilayer, a critical step in using the energy of E1 homotrimer formation to drive the fusion of the virus and target membranes. The properties of the E1 N88A,D136A mutant completely recapitulated the defect in the fusion of WT RuV in the absence of calcium, thus confirming the importance of Ca^2+^ coordination during the RuV fusion process. Together our results suggest that the primary role of RuV E1 calcium binding is not to compensate for the negatively charged D136 residue, but rather a more specific role in promoting E1-membrane interaction.

## Discussion

Here we demonstrated that Ca^2+^ is strictly required for WT RuV liposome association, membrane fusion, and virus infection. A similar specific block resulted when the key Ca^2+^ binding residues E1 N88 and/or D136 were substituted with alanine. In contrast, RuV binding to the cell surface, endocytic uptake, and E1 conformational changes did not require Ca^2+^ coordination. An early study showed an effect of calcium in cell-cell fusion mediated by HIV-1, but the mechanism of this effect is undefined [39]. Cellular calcium levels are known to be critical for infection by non-enveloped viruses such as polyomavirus and rotavirus [40], [41]. However, in these examples Ca^2+^ stabilizes the virus core and the low Ca^2+^ concentration of the cytosol promotes uncoating during virus entry. In contrast, WT RuV particles were stable in the absence of Ca^2+^ and the N88 and D136 mutants showed normal virus assembly and release. Our results thus define a specific requirement for Ca^2+^ during RuV fusion. To our knowledge, RuV E1 represents the first example of a Ca^2+^-dependent virus fusion protein.

The low pH-triggered interaction of RuV with liposomes was inhibited in the absence of Ca^2+^, reflecting the Ca^2+^-dependence of E1 insertion into the target membrane. In contrast to our results with virus particles, association of the purified RuV E1 homotrimer with target membranes occurs at either neutral or low pH in the presence of Ca^2+^, and at low pH in the absence of Ca^2+^ [28]. Although Dubois *et al.* hypothesized that calcium could be involved in direct attachment of the virus to the target cell lipid bilayer at neutral pH [28], our data demonstrated that Ca^2+^ is not required for the binding of virus to cell surface receptors, while stable E1-membrane interaction requires both low pH and Ca^2+^. The differences between our results and those of DuBois *et al.* probably reflect several differences between experimental systems, including the use of the postfusion E1 ectodomain homotrimer, overnight pH treatment, and target membranes containing negatively-charged lipids. Nevertheless, despite these differences, both studies are consistent with an important role of calcium in E1-membrane interaction.

Our data demonstrate that RuV encounters low pH and a calcium concentration compatible with fusion during virus transit through the endocytic pathway (Fig. 6E). This is intriguing because both pH and Ca^2+^ concentration are reported to fluctuate in this pathway. While the Ca^2+^ concentration is high in the extracellular milieu, one report indicates that the level of calcium drops to concentrations ≤10 µM in early endosomes [33] due to the presence of pH-dependent Ca^2+^ channels that compensate for the influx of protons into the endosomal lumen [42]. The Ca^2+^ concentration then appears to increase to 500 µM in late endosomes/lysosomes [43]. How might RuV fuse in the environment of the endocytic pathway? Our understanding of the Ca^2+^ concentration in the endocytic pathway is currently incomplete, and endosomes themselves are heterogeneous organelles with distinct characteristics [44]. The simplest explanation would be that virus-containing vesicles reach a proper pH for RuV fusion before the Ca^2+^ concentration becomes suboptimal. Alternatively, Ca^2+^ could remain bound to RuV E1 for sufficient time to promote fusion. Our data indicate that removal of calcium immediately prior to low pH treatment impaired fusion at the plasma membrane, in keeping with the relatively weak nature of Ca^2+^-E1 binding [28]. However, calcium binding might be stabilized by coordination by a lipid headgroup, replacing the acetate ion observed in the crystal structure. Alternatively, RuV might first encounter low pH in the early endosome but fuse in a later endosomal or lysosomal compartment that has a higher Ca^2+^ concentration [43], [45]. However, this model does not agree with the rapid inactivation we observed when RuV was exposed to low pH in the absence of Ca^2+^. The Ca^2+^ concentration required for virus fusion in the endosome might differ from those we observed in vitro, perhaps due to differences in lipid composition, luminal ionic environment, or membrane-associated Ca^2+^. Our limited knowledge of endosomal calcium concentration relies on imprecise probes that detect luminal calcium, while the critical concentration of calcium is presumably that at the endosomal membrane. It is even possible that the virus somehow increases the Ca^2+^ concentration of the endosome compartment, as there are examples of viruses that alter cellular calcium levels during entry [e.g., 46]. While more work will be required to define the intracellular site and Ca^2+^ conditions for RuV fusion in vivo, our data clearly demonstrate that both RuV fusion and infection require calcium.

The calcium-dependent RuV-lipid interaction is reminiscent of other calcium-dependent lipid binding proteins such as the TIM family [28], [47] and the synaptotagmin proteins [48], [49]. Bound Ca^2+^ can enable these proteins to interact with negatively-charged lipids such as phosphatidylserine [50]–[52]. However, to date our results suggest that rather than inducing binding of negatively charged lipids, Ca^2+^ plays a different role in the function of RuV E1. The liposomes used in our membrane interaction studies did not contain negatively-charged lipids, and efficient RuV-plasma membrane fusion occurred in spite of the scarcity of negatively-charged lipids in the outer leaflet of the PM [53]. E1's Ca^2+^ binding does not act simply to compensate for N88 polarity and D136 negative charge, since their substitution with apolar alanine residues did not rescue RuV membrane interaction or fusion. The two FLs are presumably optimized to form a structurally specific Ca^2+^ binding site. Indeed, while Na^+^ binds to the two FLs, Na^+^ did not permit RuV-membrane interaction or fusion. The Na^+^ bound conformation of FL2 differs significantly from that of Ca^2+^ bound FL2, including an important rotation of D136 [28]. These differences in conformation upon Ca^2+^ binding could align the two FLs in the correct orientation for optimal membrane insertion. It is also important to note that the effect of Ca^2+^ on the conformation of the FLs could differ in target membrane-inserted RuV E1 vs. the crystal structures, which were determined in solution. The calcium requirement is not simply a consequence of the presence of two FLs, since viral fusion proteins such as vesicular stomatitis virus G protein and herpes simplex virus gB also have two FLs, but function efficiently without having a metal binding site [31], [32]. Thus the mechanistic role of Ca^2+^ in RuV fusion, while clearly critical, is as yet unclear, and it will be important to address the effect of Ca^2+^ on the membrane-inserted conformation of the FLs.

To date, RuV E1 is the only viral fusion protein that is known to require metal binding for its function. Within the *Togaviviridae* and *Flaviviridae*, RuV is unique in having two FLs, a Ca^2+^ requirement, and humans as the only natural host. The selective advantage of the unique RuV fusion mechanism and the nature of the evolutionary pressure that produced it are unclear. Presumably these relate to the key step of fusion protein-membrane insertion, a process that is not well understood for any viral fusion protein. Definition of the role of Ca^2+^ in this process thus will help to advance our understanding of the overall function of viral fusion proteins and may prove useful for their therapeutic control.

## Materials and Methods

### Cells

Vero and HeLa cells from ATCC were a kind gift of Dr. Kartik Chandran, and were maintained at 37°C in growth medium: Dulbecco's modified Eagle's medium (DMEM) containing 5% fetal bovine serum, 1% L-glutamine, 100 U penicillin/ml and 100 µg streptomycin/ml. BHK-21 cells were cultured in growth medium with the addition of 10% tryptose phosphate broth. Primary human umbilical vein endothelial cells were obtained from a commercial source (Lonza, NJ) and maintained according to manufacturer's instructions in Endothelial Cell Growth Medium (Lonza, NJ).

### Infectious clones and mutagenesis

The RuV M33 infectious clone (pBRM33) [54] was a kind gift from Dr. Tom Hobman (University of Alberta, Edmonton, Canada). In vitro mutagenesis was performed using intermediate plasmids derived from pGEM-5zf(-) plasmid (Promega). pGEM33-SphI/BamHI and pGEM33-XbaI/HindIII (in which the BamHI in the multiple cloning site was removed by site-directed mutagenesis) contained the corresponding fragments from the M33 clone. pGEM33-SphI/BamHI was used as a template for circular site-directed mutagenesis [55], cut with SphI/BamHI and ligated into pGEM33-XbaI/HindIII. The mutated Xba/HindIII fragments were then subcloned into pBRM33 to generate the final RuV E1 N88A,D136A, and N88A,D136A infectious clones. Sequences were confirmed by automated DNA sequencing. The SFV strain used was a well-characterized plaque-purified isolate propagated in BHK-21 cells [56]. Purified radiolabeled SFV was derived from the pSP6-SFV4 infectious clone [57].

### In vitro transcription, virus production and titration

RuV viral RNAs were in vitro transcribed from pBRM33 and electroporated into BHK-21 cells to generate virus stocks [57]. Culture supernatants were harvested 48–72 h post-electroporation, supplemented with 10 mM Hepes pH 7, clarified by centrifugation at 1000× g for 5 min and frozen at −80°C in aliquots. RuV titers were determined by a modified infectious center assay [58]. Briefly, serial dilutions of viruses were incubated with Vero cells for 4 h at 37°C, a time point that permits maximal RuV entry, and then cultured for 48 h in the presence of 20 mM NH_4_Cl to block secondary infection. This time point was chosen because of the relatively slow replication kinetics of RuV. Infected cells were quantitated by immunofluorescence and titers calculated as infectious centers (IC)/ml.

To produce radiolabeled RuV, BHK-21 or Vero cells were electroporated with in vitro transcribed viral RNA [57]. At 24 h post-electroporation, infected cells were radiolabeled with 150 µCi/ml [S^35^]-methionine/cysteine for 24 h at 37°C. Supernatants were then harvested, clarified and pelleted at 210,000× g for 3 h at 4°C. The virus pellet was resuspended in 50 mM Tris pH 7.4, 100 mM NaCl and purified on a continuous Pfefferkorn sucrose gradient [59] and stored in aliquots at −80°C. Purified WT RuV was still infectious, reaching titers of up to 1×10^8^ IC/ml.

### Immunofluorescence

As described previously [34], cells were washed in PBS, fixed in 4% paraformaldehyde for 20 min, then permeabilized 5 min in PBS containing 0.2% Triton X-100. Permeabilized cells were washed once, then incubated 2 h at 37°C in PBS containing 5% BSA and an anti-RuV (LifeSpan BioSciences, Inc) or an anti-SFV envelope protein serum [60]. Labeled cells were washed twice, and then incubated at room temperature for 30 min in PBS containing secondary antibodies and 1 µg/ml Hoechst (Invitrogen) to counterstain nuclei. Stained cells (≥5 fields/condition) were imaged by conventional epifluorescence microscopy using a Zeiss Cell Observer system (Zeiss) equipped with an Axiovert 200M microscope and 10× objective. The proportion of infected cells was evaluated by automated counting of infected cells versus nuclei using the CellProfiler cell image analysis software (www.cellprofiler.org/). Multiplicity was adjusted to be in the linear range.

### Virus infection assays

RuV (MOI = 1) or SFV (MOI = 10) was bound on ice to pre-chilled Vero cells for 1.5 h in binding medium (RPMI without NaBicarbonate, plus 0.2% BSA and 10 mM Hepes, pH 7.0). Unbound viruses were removed by washing, and cells were shifted to 37°C to permit entry for 20 min in calcium-free media (calcium-free MEM, Sigma-Aldrich) supplemented with 10 mM Hepes pH 7, 0.2% BSA and the indicated concentrations of CaCl_2_. The cells were then incubated in growth media (with calcium) containing 20 mM NH_4_Cl for 48 h at 37°C, and infected cells were scored by immunofluorescence. Alternatively, after binding on ice the cells were shifted to 37°C for 20 min+/−2 mM CaCl_2_, followed by an additional 1 h incubation at 37°C+/− CaCl_2_ to test if infection in calcium-free media can be rescued by the subsequent addition of Ca^2+^. Cells were then cultured in growth media containing 20 mM NH_4_Cl and scored by immunofluorescence 48 h post-infection.

### Fusion-infection assay

Similar to a published assay [34], RuV (MOI 1–5) or SFV (MOI 2.5–5) was prebound for 1.5 h on ice to Vero cells in binding medium. Cells were washed and incubated at 37°C for the indicated time in fusion medium of the indicated pH (either RPMI or calcium-free MEM, without bicarbonate, plus 0.2% BSA and 20 mM MES). For experiments testing pH values lower than 5.8, fusion media was buffered with a combination of 10 mM MES and 10 mM Na-succinate. After the pH pulse cells were washed and incubated in growth medium containing 20 mM NH_4_Cl for 48 h at 37°C, and scored by immunofluorescence. To test defined concentrations of free Ca^2+^ or Mg^2+^, fusion buffer was composed of 20 mM MES, 130 mM NaCl, 5 mM glucose, 0.2% BSA and 1 mM EGTA plus added CaCl_2_ or MgCl_2_, and the concentration of free Ca^2+^ or Mg^2+^ in presence of EGTA was calculated using Webmaxc web tool (http://www.stanford.edu/~cpatton/webmaxcE.htm).

### Low-pH inactivation assay

Similar to a previously described protocol [36], RuV was spun at 4°C for 1 h at 1500× g onto poly-D-lysine treated coverslips. Coverslips with immobilized virus were washed in PBS, and treated with RPMI-based fusion medium at the indicated pH for 15 min at 37°C. A single cell suspension of Vero cells in growth medium was overlaid onto the washed coverslips and centrifuged at 200× g for 5 min. Cells were transferred to 37°C for 1 h to permit virus entry, incubated for 48 h in growth medium plus 20 mM NH_4_Cl, and scored by immunofluorescence. Note that the initial amount of virus added to the coverslips corresponded to an MOI of 1.

### Trypsin-resistance assay

The low pH-triggered conformational change in RuV E1 was quantitated by trypsin-resistance essentially as previously described [24]. In brief, purified radiolabeled RuV was treated at pH 8 or 6 in the presence of 2 mM CaCl_2_ or 1.5 mM EDTA for 5 min at 37°C. Samples were then adjusted to pH 8, solubilized with 1% NP40, and digested with 125 µg trypsin/ml for 30 min at 37°C. Digestion was quenched by addition of a final concentration of 125 µg/ml soybean trypsin inhibitor, 1 mM PMSF and 0.1 TIU aprotinin/ml. Trypsin-resistant E1 was recovered by immunoprecipitation [60] using a mAb to RuV E1 (Meridian LifeScience). Samples were boiled in LDS sample buffer under reducing conditions, resolved by SDS-PAGE and analyzed by autoradiography. The E1-E2 dimer was also assessed by immunoprecipitation of radiolabeled virus with E1 or E-specific mAbs (Meridian LifeScience).

### Virus-liposome association assay

Liposomes composed of a 1∶1∶1∶1.5 molar ratio of phosphatidylcholine (egg yolk), phosphatidylethanolamine (egg yolk), sphingomyelin (bovine brain) and cholesterol (Avanti Polar Lipids, Alabaster, Ala) were prepared in MES saline (20 mM MES pH 7; 130 mM NaCl) by freeze-thaw and extrusion through 400 nm polycarbonate filters [61]. Low pH-triggered virus-liposome association was determined based on a published method [59]. Briefly, radiolabeled RuV was mixed with 0.2 mM liposomes plus 1.5 mM EDTA or 2 mM CaCl_2_ where indicated and treated at pH 8.0 or 6.0 for 1 min at 37°C. Samples were adjusted to pH 8.0 and 40% sucrose, layered onto a 60% sucrose cushion and overlaid with 25% and 5% sucrose (all percentages are wt/vol) in 50 mM Tris pH 8 and 100 mM NaCl. Gradients were centrifuged at 210,000× g for 2 h at 4°C in the TLS-55 rotor, and fractionated into seven equal fractions. Virus-liposome co-floatation was calculated as the percentage of the total virus radioactivity recovered in the top two liposome-containing fractions. Total recovery of virus radioactivity was ∼95%.

### Lipid mixing RuV fusion assay

Purified RuV (1.2 µg) was labeled with a self-quenching concentration of the DiD lipid probe. Virus was diluted in 48 µl of buffer (5 mM HEPES, 150 mM NaCl, 0.1 mM EDTA pH 7), 2 µl 0.25 mM DiD (Invitrogen) in DMSO was injected while mildly vortexing and the mixture was incubated at room temperature for 10 min. Labeling did not cause a significant decrease in virus infectivity. Labeled particles were diluted in binding medium, and 150 ng virus was incubated for 1 h on ice with Vero cells plated in 8 well Lab-Tek chambers (Thermo Scientific, Waltham, MA). To trigger fusion at the plasma membrane, cells were then directly treated for 2 min at 37°C in fusion media at the indicated pH, supplemented with 2 mM CaCl_2_ or 2 mM EDTA. To allow fusion in endosomes, cells were washed and then incubated for 20 min at 37°C or 4°C in MEM fusion medium containing 2 mM CaCl_2_ as indicated. In parallel, 1 set of cells was pre-incubated with 20 mM NH_4_Cl for 2 h at 37°C and maintained with NH_4_Cl throughout the experiment.

Following treatments, cells were washed, fixed in 4% paraformaldehyde, and nuclei were counterstained with Hoechst. Samples were analyzed using a SP5 Leica confocal microscope equipped with an Acousto-Optical Beam Splitter (Einstein Analytical Imaging Center). Several 0.25 µm Z-stacks from several fields were taken using exactly the same settings. A maximal Z-stack projection was generated from which the DiD signal from ≥200 cells was quantified using ImageJ (NIH, http://imageJ.nih.gov/ij). The data are presented as the average amount of DiD signal calculated per nucleus enumerated.

### Growth kinetics and virus assembly assays

BHK-21 cells were electroporated with RuV RNAs, and then seeded in four 35 cm wells and cultured in growth media for 72 h. Culture medium was harvested at the indicated times and infectious particles titered by infectious center assay, all as described above. The efficiency of particle assembly was determined at 48 h post-electroporation by pelleting half of the culture medium at 210,000× g for 3 h at 4°C and lysing the cells. Proteins from both samples were resolved by SDS-PAGE and analyzed by Western-blot using an anti-RuV serum (Meridian Life Science). Virus particle release was evaluated by comparing the ratio of the virus and cell lysate E1, E2 and capsid bands using the Odyssey Infrared system (Li-Cor).

### Virus-cell binding assay

Binding of radiolabeled RuV to Vero cells was determined as previously described [59], using a 1.5 h incubation in binding medium on ice.

## Supporting Information

Figure S1 **Ca^2+^-dependence is not overcome by treatment lower than the RuV pH threshold.** Fusion-infection assays were performed with RuV in Vero cells as in Fig. 2A, but treating at the indicated pH for 4 min at 37°C in calcium-free fusion medium (control) supplemented as indicated with 1.5 mM EDTA or 2 mM CaCl_2_. Data were normalized to the pH 6.0-treated samples in medium plus CaCl_2_.
(TIF)

Figure S2 **Fusion of RuV with HeLa cells and HUVEC requires Ca^2+^.** Fusion-infection assays were performed with RuV in (A) HeLa cells or (B) HUVEC. The experiment was performed as in Fig. 2A, but treating at the indicated pH for 4 min at 37°C in calcium-free fusion medium supplemented as indicated with 1.5 mM EDTA or 2 mM CaCl_2_. Data were normalized to the pH 6.0-treated samples in medium plus CaCl_2_.
(TIF)

Figure S3 **Neither Mn^2+^ nor Zn^2+^ substitutes for Ca^2+^ in RuV fusion.** RuV fusion infection assay was performed as in Fig. 3A, in the presence of the indicated concentrations of CaCl_2_, MnCl_2_ or ZnCl_2_. Data were normalized to the pH 6.0, 2 mM CaCl_2_ sample.
(TIF)

Figure S4 **Low pH pulse does not affect adherence of RuV to culture wells.** RuV was adsorbed to poly-D-lysine coated wells and incubated for 15 min with fusion buffer of the indicated pH, as in Figure 5. Samples were then washed, fixed, permeabilized, stained and imaged by epifluorescence microscopy. Control shows culture medium from uninfected cells.
(TIF)

Figure S5 **Trypsin-resistant RuV protein species are derived from the E1 protein.** Purified radiolabeled RuV was treated at the indicated pH for 5 min at 37°C and digested with trypsin with or without inhibitor as in Figure 5C. Samples were then precipitated with mAbs to E1 or E2 and analyzed by SDS-PAGE and autoradiography.
(TIF)

Figure S6 **The E1 N88A and D136A mutations inhibit virus fusion at the plasma membrane.** A fusion infection assay was performed as described in Fig. 3A, using WT RuV (MOI = 2.5) and an equivalent volume of the RuV E1 N88A,D136A and N88A,D136A virus stocks. Fusion medium was supplemented with 2 mM CaCl_2_ (WT) or 2 and 20 mM CaCl_2_ (mutants). Graph shows the range and mean of 2 independent experiments.
(TIF)

## References

[1] Frey TK (1994) Molecular biology of rubella virus. Adv Virus Res 44: 69–160. 7817880 10.1016/S0065-3527(08)60328-0 PMC7131582

[2] Hobman T, Chantler J (2007) Rubella Virus. In: Knipe DM, Howley PM, editors. Fields Virology. 5th ed. Philadelphia, PA: Lippincott Williams and Wilkins. pp. 1069–1100.

[3] Cooper LZ (1985) The history and medical consequences of rubella. Rev Infect Dis 7 Suppl 1: S2–10. 3890105 10.1093/clinids/7.supplement_1.s2

[4] Andrus JK, de Quadros CA, Solorzano CC, Periago MR, Henderson DA (2011) Measles and rubella eradication in the Americas. Vaccine 29 Suppl 4: D91–96. 22185837 10.1016/j.vaccine.2011.04.059

[5] Measles and Rubella Initiative Annual Report. WHO Annual Report

[6] Vaheri A, von Bonsdorff CH, Vesikari T, Hovi T, Vaananen P (1969) Purification of rubella virus particles. J Gen Virol 5: 39–46. 4980900 10.1099/0022-1317-5-1-39

[7] Battisti AJ, Yoder JD, Plevka P, Winkler DC, Prasad VM, et al (2012) Cryo-electron tomography of rubella virus. J Virol 86: 11078–11085. 22855483 10.1128/JVI.01390-12 PMC3457135

[8] Mangala Prasad V, Willows SD, Fokine A, Battisti AJ, Sun S, et al (2013) Rubella virus capsid protein structure and its role in virus assembly and infection. Proc Natl Acad Sci U S A 110: 20105–20110. 24282305 10.1073/pnas.1316681110 PMC3864302

[9] Baron MD, Forsell K (1991) Oligomerization of the structural proteins of rubella virus. Virology 185: 811–819. 1962452 10.1016/0042-6822(91)90552-m

[10] Hobman TC, Lemon HF, Jewell K (1997) Characterization of an endoplasmic reticulum retention signal in the rubella virus E1 glycoprotein. J Virol 71: 7670–7680. 9311850 10.1128/jvi.71.10.7670-7680.1997 PMC192117

[11] Hobman TC, Woodward L, Farquhar MG (1992) The rubella virus E1 glycoprotein is arrested in a novel post-ER, pre-Golgi compartment. J Cell Biol 118: 795–811. 1500424 10.1083/jcb.118.4.795 PMC2289574

[12] Hobman TC, Woodward L, Farquhar MG (1995) Targeting of a heterodimeric membrane protein complex to the Golgi: rubella virus E2 glycoprotein contains a transmembrane Golgi retention signal. Mol Biol Cell 6: 7–20. 7749196 10.1091/mbc.6.1.7 PMC275811

[13] Kuhn RJ (2007) Togaviridae: The Viruses and Their Replication. In: Knipe DM, Howley PM, editors. Fields Virology. Fifth ed. Philadelphia, PA: Lippincott, Williams and Wilkins. pp. 1001–1022.

[14] Hobman TC, Lundstrom ML, Mauracher CA, Woodward L, Gillam S, et al (1994) Assembly of rubella virus structural proteins into virus-like particles in transfected cells. Virology 202: 574–585. 8030223 10.1006/viro.1994.1379

[15] Risco C, Carrascosa JL, Frey TK (2003) Structural maturation of rubella virus in the Golgi complex. Virology 312: 261–269. 12919732 10.1016/S0042-6822(03)00384-2 PMC7119121

[16] Qiu Z, Ou D, Hobman TC, Gillam S (1994) Expression and characterization of virus-like particles containing rubella virus structural proteins. J Virol 68: 4086–4091. 8189549 10.1128/jvi.68.6.4086-4091.1994 PMC236923

[17] Katow S, Sugiura A (1985) Antibody response to individual rubella virus proteins in congenital and other rubella virus infections. J Clin Microbiol 21: 449–451. 3980696 10.1128/jcm.21.3.449-451.1985 PMC271684

[18] Waxham MN, Wolinsky JS (1985) A model of the structural organization of rubella virions. Rev Infect Dis 7 Suppl 1: S133–139. 4001720 10.1093/clinids/7.supplement_1.s133

[19] Cong H, Jiang Y, Tien P (2011) Identification of the myelin oligodendrocyte glycoprotein as a cellular receptor for rubella virus. J Virol 85: 11038–11047. 21880773 10.1128/JVI.05398-11 PMC3194935

[20] Lee JY, Bowden DS (2000) Rubella virus replication and links to teratogenicity. Clin Microbiol Rev 13: 571–587. 11023958 10.1128/cmr.13.4.571-587.2000 PMC88950

[21] Kee SH, Cho EJ, Song JW, Park KS, Baek LJ, et al (2004) Effects of endocytosis inhibitory drugs on rubella virus entry into VeroE6 cells. Microbiol Immunol 48: 823–829. 15557740 10.1111/j.1348-0421.2004.tb03614.x

[22] Kielian M, Chanel-Vos C, Liao M (2010) Alphavirus entry and membrane fusion. Viruses 2: 796–825. 21546978 10.3390/v2040796 PMC3086016

[23] Petruzziello R, Orsi N, Macchia S, Rieti S, Frey TK, et al (1996) Pathway of rubella virus infectious entry into Vero cells. J Gen Virol 77(Pt 2): 303–308. 8627234 10.1099/0022-1317-77-2-303

[24] Katow S, Sugiura A (1988) Low pH-induced conformational change of rubella virus envelope proteins. J Gen Virol 69(Pt 11): 2797–2807. 3183629 10.1099/0022-1317-69-11-2797

[25] Qiu Z, Yao J, Cao H, Gillam S (2000) Mutations in the E1 hydrophobic domain of rubella virus impair virus infectivity but not virus assembly. J Virol 74: 6637–6642. 10864678 10.1128/jvi.74.14.6637-6642.2000 PMC112174

[26] Yang D, Hwang D, Qiu Z, Gillam S (1998) Effects of mutations in the rubella virus E1 glycoprotein on E1-E2 interaction and membrane fusion activity. J Virol 72: 8747–8755. 9765418 10.1128/jvi.72.11.8747-8755.1998 PMC110290

[27] Kielian M, Rey FA (2006) Virus membrane fusion proteins: more than one way to make a hairpin. Nature Reviews Microbiology 4: 67–76. 16357862 10.1038/nrmicro1326 PMC7097298

[28] DuBois RM, Vaney MC, Tortorici MA, Kurdi RA, Barba-Spaeth G, et al (2013) Functional and evolutionary insight from the crystal structure of rubella virus protein E1. Nature 493: 552–556. 23292515 10.1038/nature11741

[29] Harrison SC (2008) Viral membrane fusion. Nat Struct Mol Biol 15: 690–698. 18596815 10.1038/nsmb.1456 PMC2517140

[30] Kadlec J, Loureiro S, Abrescia NG, Stuart DI, Jones IM (2008) The postfusion structure of baculovirus gp64 supports a unified view of viral fusion machines. Nat Struct Mol Biol 15: 1024–1030. 18776902 10.1038/nsmb.1484

[31] Heldwein EE, Lou H, Bender FC, Cohen GH, Eisenberg RJ, et al (2006) Crystal structure of glycoprotein B from herpes simplex virus 1. Science 313: 217–220. 16840698 10.1126/science.1126548

[32] Roche S, Bressanelli S, Rey FA, Gaudin Y (2006) Crystal structure of the low-pH form of the vesicular stomatitis virus glycoprotein G. Science 313: 187–191. 16840692 10.1126/science.1127683

[33] Gerasimenko JV, Tepikin AV, Petersen OH, Gerasimenko OV (1998) Calcium uptake via endocytosis with rapid release from acidifying endosomes. Curr Biol 8: 1335–1338. 9843688 10.1016/s0960-9822(07)00565-9

[34] Liao M, Kielian M (2005) Domain III from class II fusion proteins functions as a dominant-negative inhibitor of virus membrane fusion. J Cell Biol 171: 111–120. 16216925 10.1083/jcb.200507075 PMC2171229

[35] White J, Helenius A (1980) pH-dependent fusion between the Semliki Forest virus membrane and liposomes. Proc Natl Acad Sci U S A 77: 3273–3277. 6997876 10.1073/pnas.77.6.3273 PMC349597

[36] Sharma NR, Mateu G, Dreux M, Grakoui A, Cosset FL, et al (2011) Hepatitis C virus is primed by CD81 protein for low pH-dependent fusion. J Biol Chem 286: 30361–30376. 21737455 10.1074/jbc.M111.263350 PMC3162395

[37] Liu CY, Kielian M (2009) E1 mutants identify a critical region in the trimer interface of the Semliki forest virus fusion protein. J Virol 83: 11298–11306. 19692469 10.1128/JVI.01147-09 PMC2772772

[38] White JM, Delos SE, Brecher M, Schornberg K (2008) Structures and mechanisms of viral membrane fusion proteins: multiple variations on a common theme. Crit Rev Biochem Mol Biol 43: 189–219. 18568847 10.1080/10409230802058320 PMC2649671

[39] Dimitrov DS, Broder CC, Berger EA, Blumenthal R (1993) Calcium ions are required for cell fusion mediated by the CD4-human immunodeficiency virus type 1 envelope glycoprotein interaction. Journal of virology 67: 1647–1652. 8437234 10.1128/jvi.67.3.1647-1652.1993 PMC237536

[40] Haynes JI 2nd, Chang D, Consigli RA (1993) Mutations in the putative calcium-binding domain of polyomavirus VP1 affect capsid assembly. J Virol 67: 2486–2495. 8386264 10.1128/jvi.67.5.2486-2495.1993 PMC237567

[41] Dormitzer PR, Greenberg HB, Harrison SC (2000) Purified recombinant rotavirus VP7 forms soluble, calcium-dependent trimers. Virology 277: 420–428. 11080489 10.1006/viro.2000.0625

[42] Saito M, Hanson PI, Schlesinger P (2007) Luminal chloride-dependent activation of endosome calcium channels: patch clamp study of enlarged endosomes. J Biol Chem 282: 27327–27333. 17609211 10.1074/jbc.M702557200

[43] Christensen KA, Myers JT, Swanson JA (2002) pH-dependent regulation of lysosomal calcium in macrophages. J Cell Sci 115: 599–607. 11861766 10.1242/jcs.115.3.599

[44] Lakadamyali M, Rust MJ, Zhuang X (2006) Ligands for clathrin-mediated endocytosis are differentially sorted into distinct populations of early endosomes. Cell 124: 997–1009. 16530046 10.1016/j.cell.2005.12.038 PMC2660893

[45] Lloyd-Evans E, Morgan AJ, He X, Smith DA, Elliot-Smith E, et al (2008) Niemann-Pick disease type C1 is a sphingosine storage disease that causes deregulation of lysosomal calcium. Nat Med 14: 1247–1255. 18953351 10.1038/nm.1876

[46] Nour AM, Li Y, Wolenski J, Modis Y (2013) Viral membrane fusion and nucleocapsid delivery into the cytoplasm are distinct events in some flaviviruses. PLoS pathogens 9: e1003585. 24039574 10.1371/journal.ppat.1003585 PMC3764215

[47] Santiago C, Ballesteros A, Tami C, Martinez-Munoz L, Kaplan GG, et al (2007) Structures of T Cell immunoglobulin mucin receptors 1 and 2 reveal mechanisms for regulation of immune responses by the TIM receptor family. Immunity 26: 299–310. 17363299 10.1016/j.immuni.2007.01.014 PMC7111020

[48] Fukuda M, Kojima T, Mikoshiba K (1996) Phospholipid composition dependence of Ca2+-dependent phospholipid binding to the C2A domain of synaptotagmin IV. J Biol Chem 271: 8430–8434. 8626542 10.1074/jbc.271.14.8430

[49] Martens S, McMahon HT (2008) Mechanisms of membrane fusion: disparate players and common principles. Nat Rev Mol Cell Biol 9: 543–556. 18496517 10.1038/nrm2417

[50] Herrick DZ, Sterbling S, Rasch KA, Hinderliter A, Cafiso DS (2006) Position of synaptotagmin I at the membrane interface: cooperative interactions of tandem C2 domains. Biochemistry 45: 9668–9674. 16893168 10.1021/bi060874j

[51] Hui E, Bai J, Chapman ER (2006) Ca2+-triggered simultaneous membrane penetration of the tandem C2-domains of synaptotagmin I. Biophys J 91: 1767–1777. 16782782 10.1529/biophysj.105.080325 PMC1544279

[52] DeKruyff RH, Bu X, Ballesteros A, Santiago C, Chim YL, et al (2010) T cell/transmembrane, Ig, and mucin-3 allelic variants differentially recognize phosphatidylserine and mediate phagocytosis of apoptotic cells. J Immunol 184: 1918–1930. 20083673 10.4049/jimmunol.0903059 PMC3128800

[53] van Meer G, Voelker DR, Feigenson GW (2008) Membrane lipids: where they are and how they behave. Nat Rev Mol Cell Biol 9: 112–124. 18216768 10.1038/nrm2330 PMC2642958

[54] Yao J, Gillam S (1999) Mutational analysis, using a full-length rubella virus cDNA clone, of rubella virus E1 transmembrane and cytoplasmic domains required for virus release. J Virol 73: 4622–4630. 10233921 10.1128/jvi.73.6.4622-4630.1999 PMC112503

[55] Fontana J, Lopez-Iglesias C, Tzeng WP, Frey TK, Fernandez JJ, et al (2010) Three-dimensional structure of Rubella virus factories. Virology 405: 579–591. 20655079 10.1016/j.virol.2010.06.043 PMC7111912

[56] Glomb-Reinmund S, Kielian M (1998) fus-1, a pH shift mutant of Semliki Forest virus, acts by altering spike subunit interactions via a mutation in the E2 subunit. J Virol 72: 4281–4287. 9557718 10.1128/jvi.72.5.4281-4287.1998 PMC109658

[57] Liljestrom P, Lusa S, Huylebroeck D, Garoff H (1991) In vitro mutagenesis of a full-length cDNA clone of Semliki Forest virus: the small 6,000-molecular-weight membrane protein modulates virus release. J Virol 65: 4107–4113. 2072446 10.1128/jvi.65.8.4107-4113.1991 PMC248843

[58] Umashankar M, Sanchez-San Martin C, Liao M, Reilly B, Guo A, et al (2008) Differential cholesterol binding by class II fusion proteins determines membrane fusion properties. J Virol 82: 9245–9253. 18632857 10.1128/JVI.00975-08 PMC2546879

[59] Chanel-Vos C, Kielian M (2004) A conserved histidine in the ij loop of the Semliki Forest virus E1 protein plays an important role in membrane fusion. J Virol 78: 13543–13552. 15564465 10.1128/JVI.78.24.13543-13552.2004 PMC533937

[60] Kielian M, Jungerwirth S, Sayad KU, DeCandido S (1990) Biosynthesis, maturation, and acid activation of the Semliki Forest virus fusion protein. J Virol 64: 4614–4624. 2118964 10.1128/jvi.64.10.4614-4624.1990 PMC247945

[61] Chatterjee PK, Vashishtha M, Kielian M (2000) Biochemical consequences of a mutation that controls the cholesterol dependence of Semliki Forest virus fusion. J Virol 74: 1623–1631. 10644331 10.1128/jvi.74.4.1623-1631.2000 PMC111636

