# Supplementary figures and images for: Rubella Virus: First Calcium-Requiring Viral Fusion Protein
Source: PLoS Pathog. 2014 Dec 4;10(12):e1004530. doi: 10.1371/journal.ppat.1004530 (PMC4256232; doi:10.1371/journal.ppat.1004530)

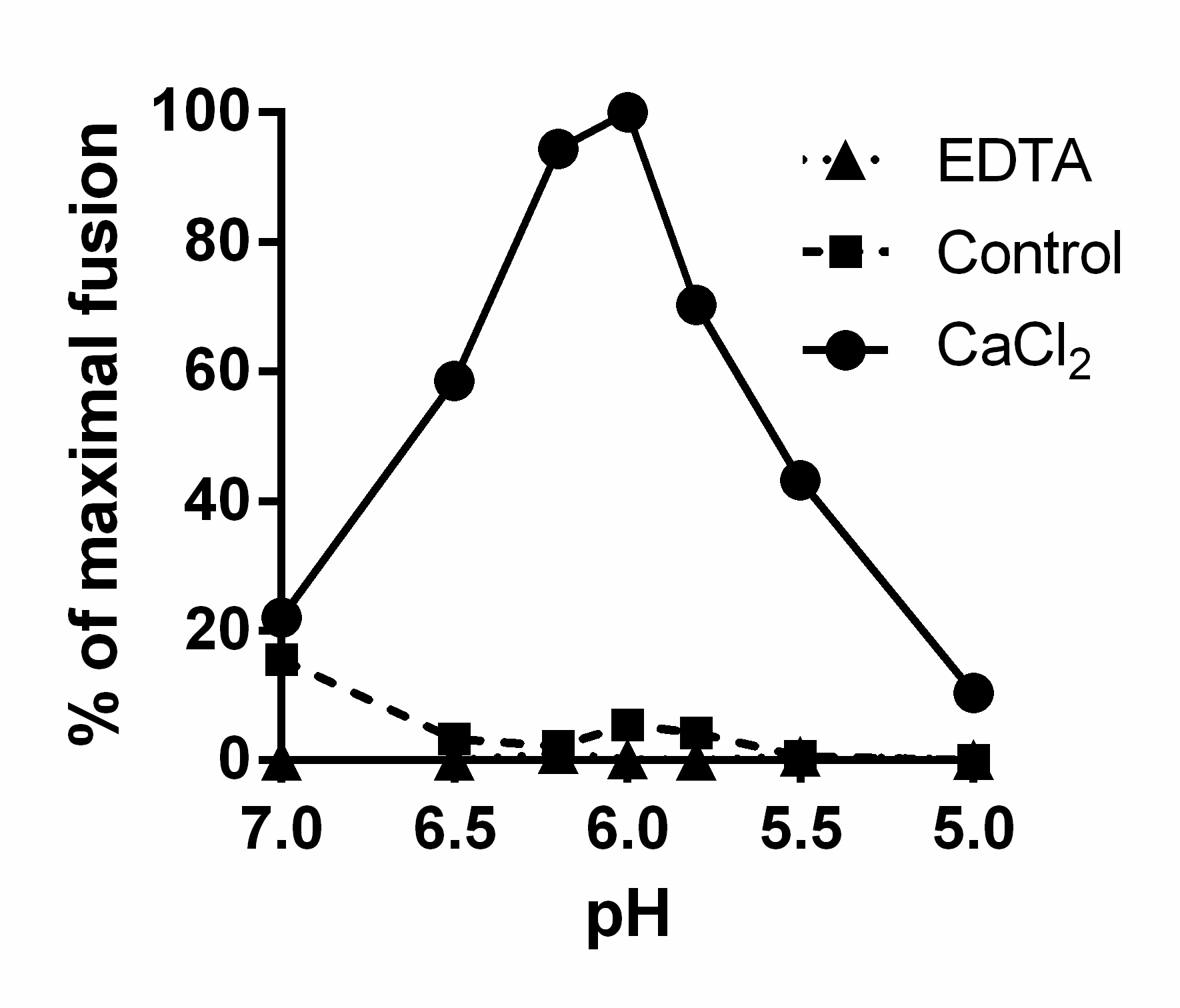

Supplement: Figure S1 — Ca2+-dependence is not overcome by treatment lower than the RuV pH threshold. Fusion-infection assays were performed with RuV in Vero cells as in Fig. 2A, but treating at the indicated pH for 4 min at 37°C in calcium-free fusion medium (control) supplemented as indicated with 1.5 mM EDTA or 2 mM CaCl2. Data were normalized to the pH 6.0-treated samples in medium plus CaCl2. (TIF) [file ppat.1004530.s001.tif]

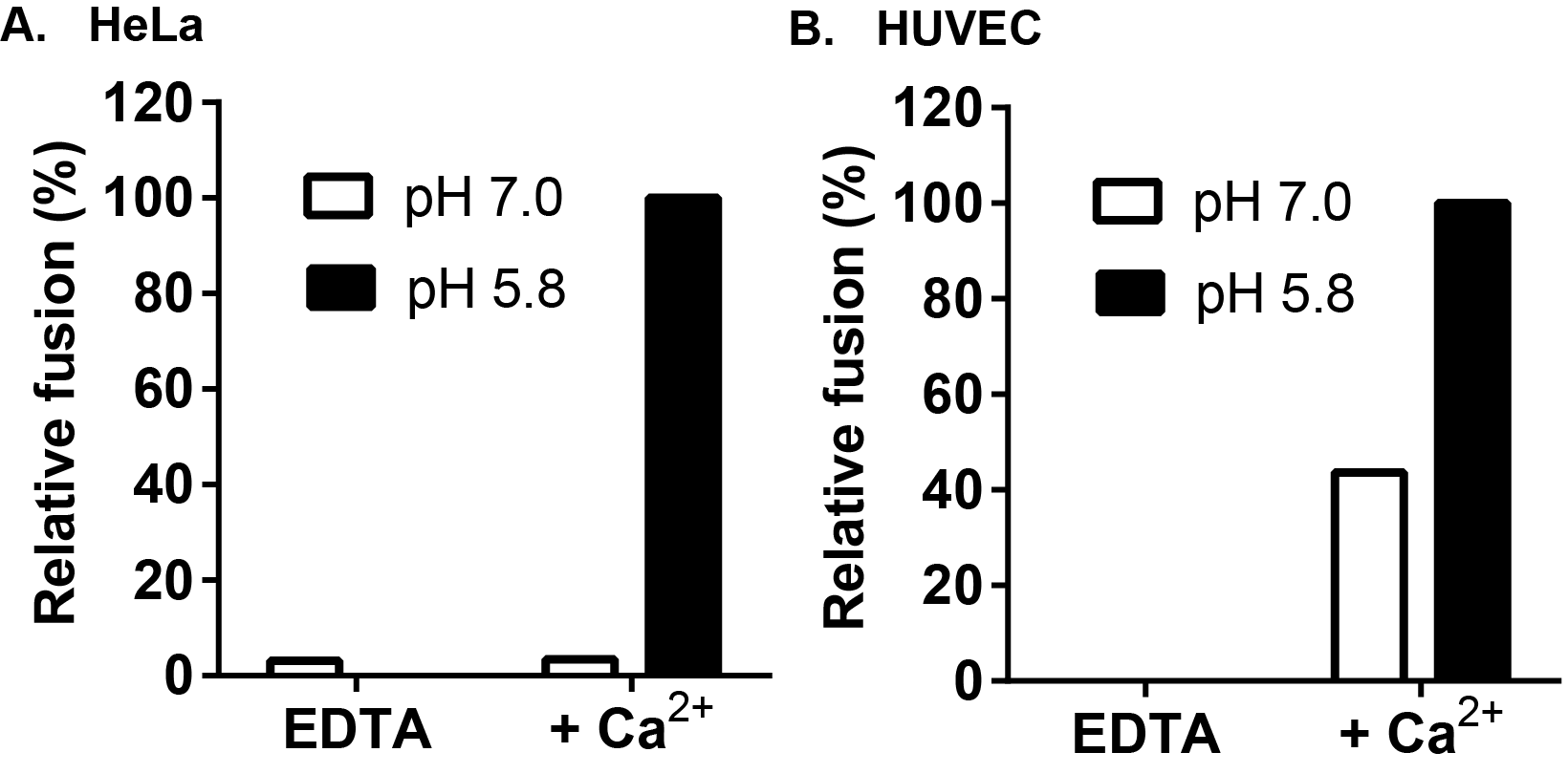

Supplement: Figure S2 — Fusion of RuV with HeLa cells and HUVEC requires Ca2+. Fusion-infection assays were performed with RuV in (A) HeLa cells or (B) HUVEC. The experiment was performed as in Fig. 2A, but treating at the indicated pH for 4 min at 37°C in calcium-free fusion medium supplemented as indicated with 1.5 mM EDTA or 2 mM CaCl2. Data were normalized to the pH 6.0-treated samples in medium plus CaCl2. (TIF) [file ppat.1004530.s002.tif]

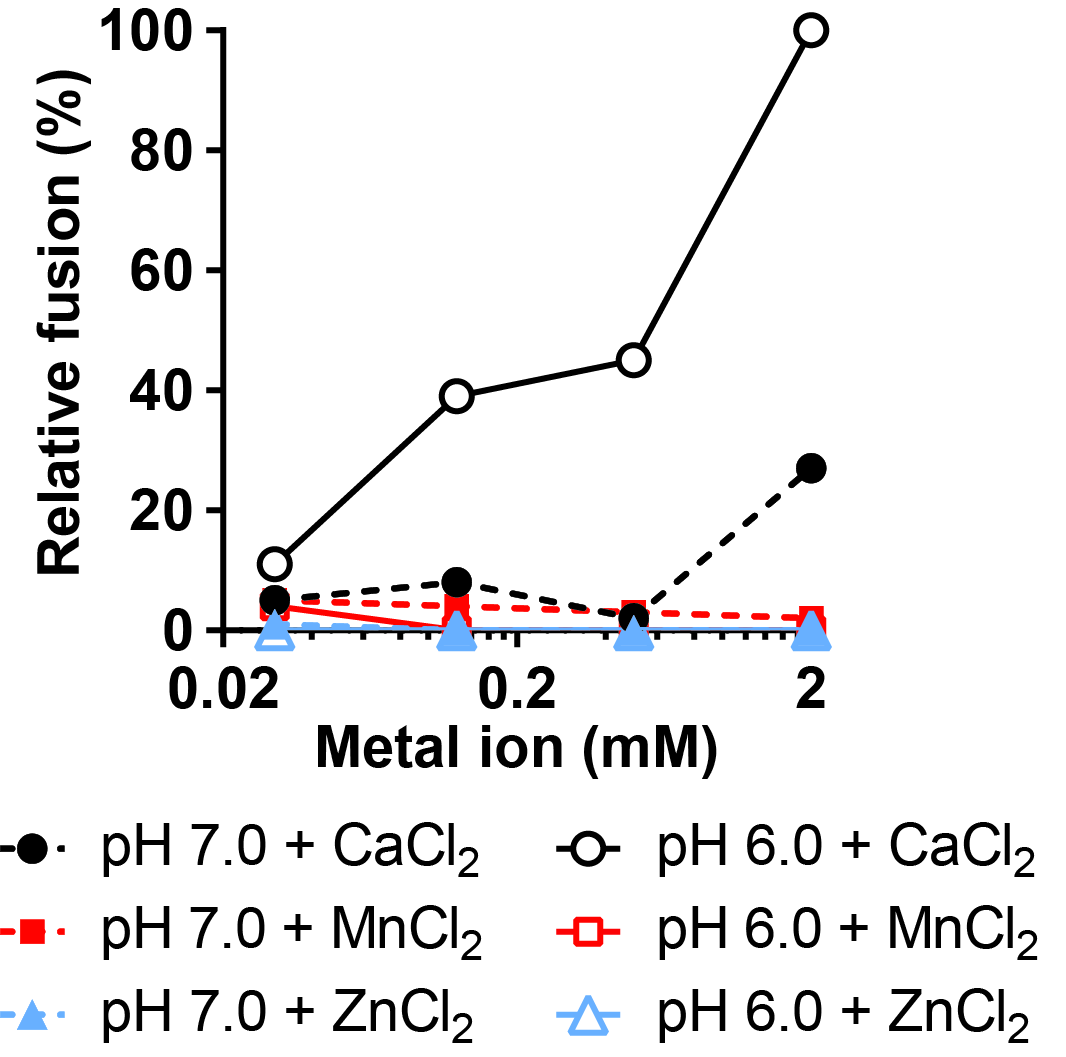

Supplement: Figure S3 — Neither Mn2+ nor Zn2+ substitutes for Ca2+ in RuV fusion. RuV fusion infection assay was performed as in Fig. 3A, in the presence of the indicated concentrations of CaCl2, MnCl2 or ZnCl2. Data were normalized to the pH 6.0, 2 mM CaCl2 sample. (TIF) [file ppat.1004530.s003.tif]

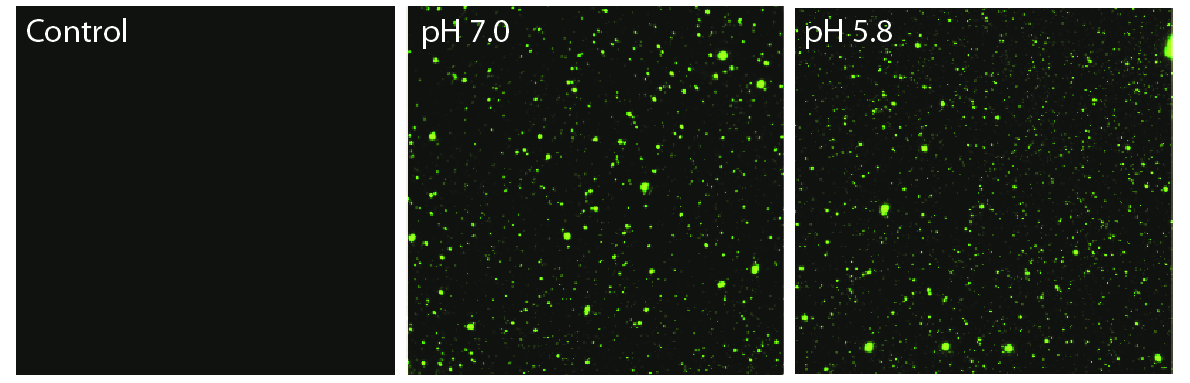

Supplement: Figure S4 — Low pH pulse does not affect adherence of RuV to culture wells. RuV was adsorbed to poly-D-lysine coated wells and incubated for 15 min with fusion buffer of the indicated pH, as in Figure 5. Samples were then washed, fixed, permeabilized, stained and imaged by epifluorescence microscopy. Control shows culture medium from uninfected cells. (TIF) [file ppat.1004530.s004.tif]

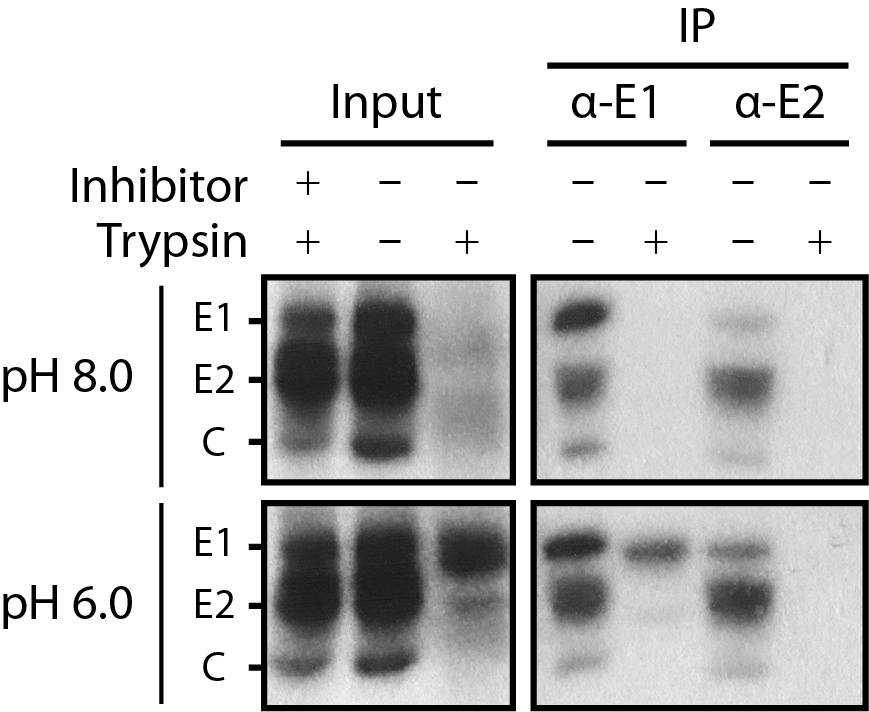

Supplement: Figure S5 — Trypsin-resistant RuV protein species are derived from the E1 protein. Purified radiolabeled RuV was treated at the indicated pH for 5 min at 37°C and digested with trypsin with or without inhibitor as in Figure 5C. Samples were then precipitated with mAbs to E1 or E2 and analyzed by SDS-PAGE and autoradiography. (TIF) [file ppat.1004530.s005.tif]

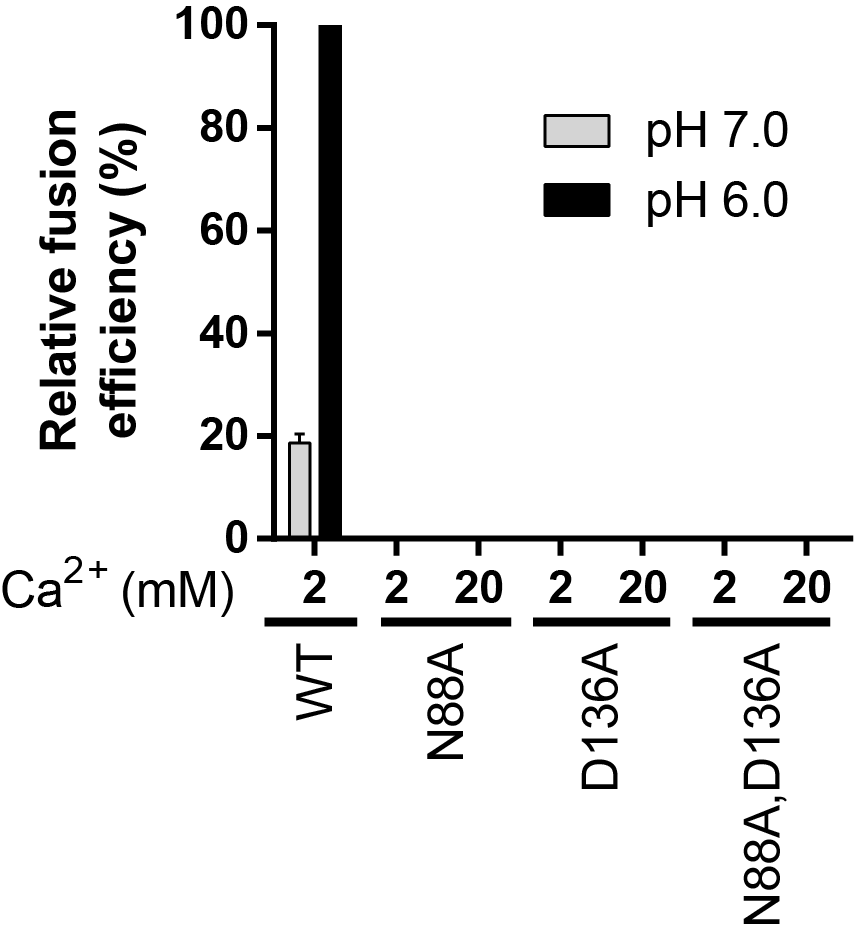

Supplement: Figure S6 — The E1 N88A and D136A mutations inhibit virus fusion at the plasma membrane. A fusion infection assay was performed as described in Fig. 3A, using WT RuV (MOI = 2.5) and an equivalent volume of the RuV E1 N88A,D136A and N88A,D136A virus stocks. Fusion medium was supplemented with 2 mM CaCl2 (WT) or 2 and 20 mM CaCl2 (mutants). Graph shows the range and mean of 2 independent experiments. (TIF) [file ppat.1004530.s006.tif]
